# Random Forests Are Able to Identify Differences in Clotting Dynamics from Kinetic Models of Thrombin Generation

**DOI:** 10.1371/journal.pone.0153776

**Published:** 2016-05-12

**Authors:** Jayavel Arumugam, Satish T. S. Bukkapatnam, Krishna R. Narayanan, Arun R. Srinivasa

**Affiliations:** 1 Department of Mechanical Engineering, Texas A&M University, College Station, Texas, United States of America; 2 Department of Industrial and Systems Engineering, Texas A&M University, College Station, Texas, United States of America; 3 Department of Electrical and Computer Engineering, Texas A&M University, College Station, Texas, United States of America; Kurume University School of Medicine, JAPAN

## Abstract

Current methods for distinguishing acute coronary syndromes such as heart attack from stable coronary artery disease, based on the kinetics of thrombin formation, have been limited to evaluating sensitivity of well-established chemical species (e.g., thrombin) using simple quantifiers of their concentration profiles (e.g., maximum level of thrombin concentration, area under the thrombin concentration versus time curve). In order to get an improved classifier, we use a 34-protein factor clotting cascade model and convert the simulation data into a high-dimensional representation (about 19000 features) using a piecewise cubic polynomial fit. Then, we systematically find plausible assays to effectively gauge changes in acute coronary syndrome/coronary artery disease populations by introducing a statistical learning technique called Random Forests. We find that differences associated with acute coronary syndromes emerge in combinations of a handful of features. For instance, concentrations of 3 chemical species, namely, active alpha-thrombin, tissue factor-factor VIIa-factor Xa ternary complex, and intrinsic tenase complex with factor X, at specific time windows, could be used to classify acute coronary syndromes to an accuracy of about 87.2%. Such a combination could be used to efficiently assay the coagulation system.

## Introduction

In the United States, heart diseases were the leading account for death in the twentieth century and continue to be so in the twenty-first century [1]. Identifying patients at risk of acute coronary syndromes (ACS) and predicting probable courses of disease could help provide timely medical intervention; understanding the physiology of the diseases in patient-specific terms could also help design better drugs and monitor treatment more effectively. Current efforts towards patient-specific characterization include differentiating systemic changes to blood coagulation in ACS from coronary artery disease (CAD) populations [2]. Blood is observed in a hyper-coagulable state after ACS [3]. The nature or extent of the hyper-coagulability, as well as its relation to and its presence before the acute condition are not well understood. This could be attributed to at least two reasons: i) lack of assays to efficiently and effectively determine the status of blood chemistry [4]; and ii) lack of adequate statistical and mathematical tools to understand blood coagulation system involving large numbers of variables.

In the last two decades, sustained interest has been shown in empirical and computational thrombin generation assays [5] to study the coagulation system under abnormal conditions. Brummel-Ziedens et al. [6] studied alterations in thrombin dynamics between ACS and CAD. Features of thrombin profile like maximum value, area under the curve, and maximum rate were higher in ACS than CAD, suggesting hyper-coagulability. Recently there have been attempts to study changes in factor Xa (fXa), in another hyper-coagulable condition—deep vein thrombosis, using computational models [7]. Features similar to those used for thrombin were used to describe fXa. We have good prior knowledge about thrombin and fXa, which are both active chemical species that play significant roles in clotting. Naturally, the following questions arise:

1. Do the dynamics of any other chemical species change significantly?
2. Are there better features to characterize changes in the system?
3. Can we efficiently assay the entire system without losing much information pertaining to classification?

We seek answers to these questions using simulations. We study blood coagulation using a model for the Tissue factor(Tf)-initiated extrinsic pathway developed by Hockin et al. [8]. The model uses a system of ordinary nonlinear differential equations to describe dynamics of thrombin evolution. The model has copious empirical validation and has been previously used for risk analyses between ACS/CAD [6]. The number of chemical species involved is large (34 in this case), and their responses are varied, typically requiring large numbers of features to represent the time profiles.

We use a non-parametric statistical learning algorithm—Random Forests [9] to classify ACS and CAD populations. Random Forests can be used to capture highly nonlinear class boundaries, and is robust to outliers in data and to lots of noisy features. Random Forests technique allows us to filter significant species and find their critical aspects. Moreover, unlike the current use of isolated features for group comparisons prevalent in thrombin generation literature [5], use of Random Forests here exploits the role that interactions of features play in order to classify data into various groups.

Our objective is to find a small combination of features (localized regions in the state space of the model, and labelled in time) which discriminate ACS and CAD well.

## Methods

### Clotting Simulations for 3600 Seconds, Initiated with 5 pM Tf

We carried out the simulations using the Tf-initiated clotting model [8]. In the simulations, clotting was initiated with 5 pM trigger Tf. To get the initial condition data for the clotting model, we used reported mean and standard deviation data of the procoagulant and anticoagulant factor percentages in ACS and CAD populations [6]. We sampled 200 sets of these positive nonzero percentage values for each group (ACS and CAD) from lognormal distributions (data provided in S1 Dataset). These percentage values were scaled using the physiological mean values [8] (1.4E-06 M for factor II, 2.0E-08 M for factor V, 1.0E-08 M for factor VII, 7.0E-10 M for factor VIII, 9.0E-08 M for factor IX, 1.6E-07 M for factor X, 2.5E-09 M for tissue factor pathway inhibitor, and 3.4E-06 M for antithrombin). Solution profiles for all chemical species were obtained for 3600 seconds using MATLAB’s (MATLAB 8.5.0, The MathWorks, Inc., Natick, Massachusetts, United States) *‘ode15s’* stiff solver.

### Piecewise Polynomial Representation of Simulation Profiles

We normalized the simulation profiles by their respective physiological mean peak values and fit them with piecewise cubic hermite interpolating polynomials (PCHIP) [10] using MATLAB’s *‘pchip’* function. Data in each profile was divided into pieces (time intervals), and a cubic polynomial was fit in each piece while ensuring smoothness across pieces. PCHIP technique ensured the resulting interpolation changed monotonically in each interval, thereby avoiding spurious oscillations inherent in a regular spline interpolation. We used 139 pieces—each of length approximately 26 seconds. This captured fast changes, such as the time it takes for Tf-fVIIa to reach its first peak since addition of the trigger, reasonably well. PCHIP representation serves two purposes: i) it efficiently stores large amounts of simulation data; and ii) since the polynomials represent data well, the coefficients of the polynomials could act as features for classification.

### Feature Extraction from Simulation Profiles

The central idea of the scheme is to consider the data points (simulation profiles) as a “noisy-image” in a very high-dimensional space from which we try to extract features with semantic attributes like “concentration is high”, “concentration profile is sharply curved”, etc. This was implemented by extracting different kinds of features and using them in the classification study to characterize the system. In order to capture the dynamics of each species at different times during the simulation, we use the PCHIP coefficients as features. Since there is a lack of classification study to compare this work with, we used classification results of the plasma factor composition (initial conditions data used for the simulation), and the features that are conventionally studied to compare with the performance of PCHIP features considered here. Moreover, we study a fourth set of features which have the possibility of direct experimental observation.

The list of features we extracted and used for classification include the following four sets:

1. **PCHIP features to characterize dynamics**—this set includes 18904 PCHIP coefficients obtained during data representation. This set uses two datasets [6, 8] as described at the beginning of the section Methods. For a given species, there are 4 coefficients in each time interval. The coefficients are such that the fit polynomial in an interval starting at *t*_*i*_ has the form *C*_*i*3_(*t* − *t*_*i*_)^3^ + *C*_*i*2_(*t* − *t*_*i*_)^2^ + *C*_*i*1_(*t* − *t*_*i*_) + *C*_*i*0_. These coefficients have information pertaining to function values and derivatives up to 3 orders at time *t*_*i*_. Information in second and third derivatives is expected to be weak as PCHIP enforces monotonicity. Variables corresponding to the two forms of thrombin, IIa (alpha-thrombin) and mIIa (meizothrombin), were interpolated separately.
2. **Plasma factor composition**—this set consists of 8 non-zero initial condition percentage values of procoagulant and anticoagulant factors used for model simulations [6] (S1 Dataset).
3. **Conventional features**—this set consists of 11 features used to characterize active thrombin [6] and fXa profiles [7]. This includes time to reach 2 nM (for active thrombin), area under the curve for active thrombin and fXa, maximum level reached by active thrombin and fXa, maximum rate in active thrombin and fXa profiles, time to reach those maximum levels for active thrombin and fXa, and time to reach maximum rates for active thrombin and fXa. Data from two datasets [6, 8] are used in this set.
4. **Moving averages of concentration values**—this set consists of 200-second moving average (200s-MA) features extracted at uniform time intervals. For each chemical species, we extracted these 18 time-averaged function values of simulation profiles ($1/200\int_{t}^{t+200}x\left(t\right)dt$, where *x*(*t*) is concentration of a given species) at every 200 seconds starting at 100 seconds. 612 such features were extracted from all species (18 each for 34 species). This set makes use of two datasets [6, 8]. These features localize significance of each species in a time frame of about 3 minutes. Moreover, averaging over time gives a more robust feature with respect to time lags and noise imposed by model and model parameters.

These features are used as inputs in the Random Forests classification algorithm, which outputs group identity (ACS/CAD).

### ACS/CAD Classification using Random Forests

The core objective of any classification method is to label a collection of data/measurements using certain features [11]. Here we use Random Forests [9] which is formed by aggregating an ensemble of decision trees [12].

#### Decision Tree

A decision tree [12–14] divides the feature space into a number of non-overlapping regions. The regions have an equivalent tree representation in which each node is a decision rule regarding class identity. Such trees are nonparameteric and assume no particular form of the data. The task of the tree algorithm is to frame decision rules that suit the data. Such decision rules are invariant to all monotone transformations in the data [12]. Once a tree is formed, data points with unknown classes are assigned a class based on these decision rules. Decision trees have been used in the study of thrombin generation systems [15].

However, a simple tree structure is sensitive to perturbations in the data [16] which could propagate down the tree and lead to very different class labels. The random forest technique [9, 17], which uses an ensemble of trees and aggregates the results, offers a solution to this problem.

#### Random Forests

In Random Forests, the learning process of each tree involves two types of random subset selection. First, each tree in the ensemble is built with a random subset of the training data. The other subset which is kept ‘out’ is called as the out-of-bag (OOB) samples. These OOB samples are used for finding internal estimates such as error rates. Second, each decision rule in a tree is made only using a random subset of all features. This avoids the classification results being unduly biased by a few sensitive features most of the time. Such classification results aggregated from many trees can capture complex and highly nonlinear class boundaries. It is well known [18] that the method avoids overfitting of the training data, a feature which is vital when there is limited or scarce data.

Random Forests methods are known to perform well in a variety of fields such as in gene selection in microarray data [19], and in functional studies of chemical compounds [20]. In empirical studies, Random Forests compares well with other classification algorithms [21, 22], and performs consistently well in high-dimensions [23]. Use of Random Forests in clinical studies include study of blood proteins in Alzeimer’s disease [24, 25].

A key feature of the Random Forests approach is their ability to provide reliable internal estimates to monitor error rates, and it has sharp measures to rank significance of features. In particular, we made use of OOB error rate and mean decrease in Gini index (MDGini) (see below). Since this error rate does not involve data used in training a given tree, using this error rate provides inherent cross validation [26].

#### OOB Error Rate

OOB samples are used to find error rates for each tree in the ensemble, and all such error rates are averaged to get the OOB error rate. Empirical studies suggest OOB error rates are good estimates for generalization error [9, 26]. We used OOB error rates to assess the accuracy of the classifiers which are reported as percentages of (1.0 − OOB error rate).

#### Feature Significance Measure—MDGini

Feature significance was interpreted using a Random Forest importance measure known as ‘Mean Decrease in Gini index’ [27]. Typically, the decision rules in the trees are not pure in the sense that the corresponding region in feature space is heterogeneous; i.e., there is a mix of data points from all classes (in our case 2). Gini index (or Gini impurity) [16] for a decision rule is a measure of this mix; it is zero only when the decision rule is perfect (the region is homogenous). It is maximum when the mix is the highest (half-and-half mix from both the classes).

**Fig 1:**
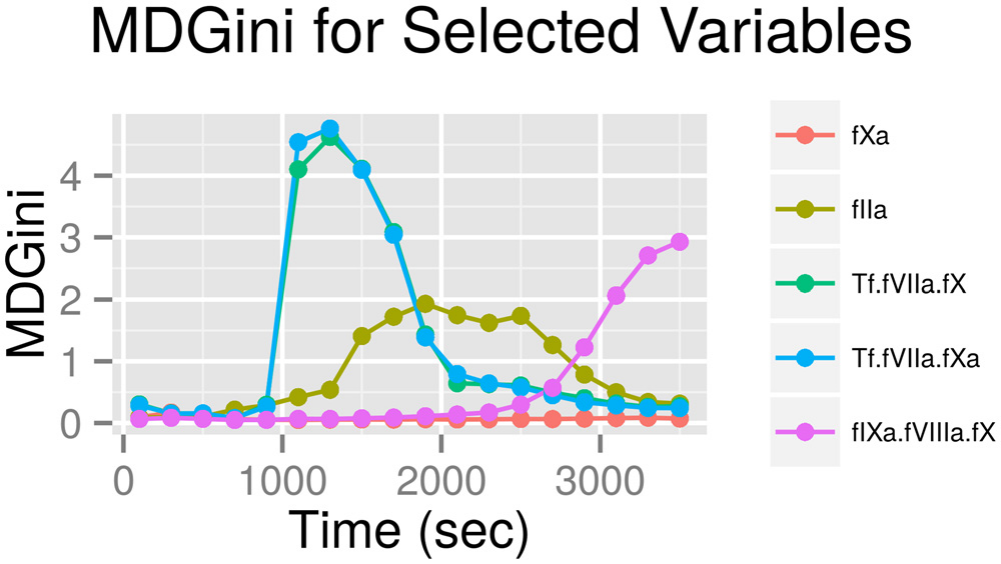
MDGini variation (in the classifier built with 200s-MA features) with time for the five selected chemical species. Tf-fVIIa-fXa and Tf-fVIIa-fX are most significant during 1000–1600 seconds, and IIa during 1400–2500 seconds from the addition of the trigger. Significance of fIXa-fVIIIa-fX increases monotonically and remains most significant at 3600 seconds suggesting that it is a long-lived species. 200s-MA—200-second moving average; MDGini—Mean Decrease in Gini index; Tf-fVIIa-fXa—Tissue factor-factor VIIa-factor Xa; Tf-fVIIa-fX—Tissue factor-factor VIIa-factor X; fIXa-fVIIIa-fX—factor IXa-factor VIIIa-factor X; IIa—activated alpha-thrombin.

**Fig 2:**
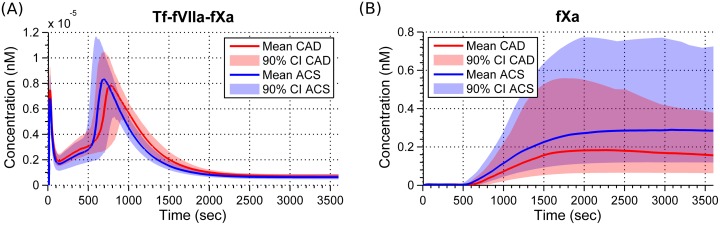
Means and 90% quantiles for Tf-fVIIa-fXa and fXa simulation profiles in ACS and CAD populations. A: Tf-fVIIa-fXa concentration profiles from the two groups split significantly from about 1000 to 1500 seconds. B: In fXa concentration profiles, there is a huge variation in both ACS and CAD populations. However, the profiles from the two groups overlap and make the features of this species weak for classification. Tf-fVIIa-fXa—Tissue factor-factor VIIa-factor Xa; fXa—factor Xa.

MDGini involves randomly permuting OOB sample data corresponding to the decision rule in a tree, and estimating the change (decrease) in Gini index. If the decrease is high while perturbing a feature, it suggests that the classification is highly dependent on that particular feature. This provides an information-theoretic feature significance measure. It inherits the invariance property of the decision rules, i.e., absolute values of the features do not matter. This is a very sharp feature significance measure (see figures 1, 2 and 6 in [28]). We use MDGini here to find even minute differences that are significant between ACS/CAD.

We used the ‘randomForest’ package in R [29] for our analysis. For each Random Forest classifier, 501 trees are used in the ensemble. To account for statistical variation between runs, we report mean and standard deviation (SD) of classification accuracies based on 50 runs.

## Results

### Classification Performance of the Entire System

Classification using initial factors has a mean accuracy of 88.13% (Table 1). Conventional features of fXa and active thrombin classify with lower mean accuracies, 82.58% and 81.04%, respectively. Using all PCHIP coefficients and all 200s-MA values result in classification accuracies of 88.59% and 88.78% respectively, which are slightly better than using 8 initial factors. At this point, one might wonder if combinations of initial conditions suffice to characterize the system. However, we note that the same set of initial conditions could give different dynamics if the reaction network is perturbed (say, rate constants are changed due to a drug or a mutated form of a coagulation factor). Hence, studying initial conditions might not suffice to characterize the dynamics of the system. Moreover, studying the dynamics of chemical species gives more physiological insight about the underlying process.

**Table 1:** Classification accuracies (%) of different sets of features. PCHIP and moving average features classify better than conventional parameters, and slightly better than all nonzero initial conditions.

Random Forest Classifier	Mean (SD)
PCHIP Features	
All PCHIP Coefficients	88.59 (0.36)
Plasma Factor Composition	
8 Initial Conditions	88.13 (0.49)
Conventional Features	
fXa	82.58 (0.53)
Active Thrombin	81.04 (0.46)
Moving Averages	
All 200s-MA	88.78 (0.32)

fXa—factor Xa; PCHIP—piecewise cubic hermite interpolating polynomials; 200s-MA—200-second moving average.

Classification accuracies quantify the information in various features with respect to ACS/CAD classification. Although, minimum and maximum accuracies in Table 1 vary over a small range (∼ 81%-89%), they offer a potent way to compare features and quantify relevant differences. Loss of 11% accuracy in the initial conditions classifier is due to the overlap in the initial condition data used (though the means were significantly different for prothrombin, factor VIII, tissue factor pathway inhibitor, and antithrombin [6], the samples from lognormal distributions used for this study overlapped). Also, the best possible accuracy is restricted by the choice of features.

### Selection of a List of Significant Species

We robustly selected a list of species that behave differently in ACS/CAD. We based our selection heuristics on three criteria and selected five species:

1. fXa and IIa were selected due to their known significance.
2. Tf-fVIIa-fXa, Tf-fVII-fX—these species had high averages for MDGini values in the classifier built with all PCHIP coefficients. MDGini values for each species were sorted and the highest ∼10% of the values were used for selection criterion. Use of just one of the highest MDGini value for each species would be too biased and prone to noise; use of all MDGini values caused huge variation in the values, blurring out differences between species.
3. fIXa-fVIIIa-fX—this species had the highest significance during the last 600 seconds of the simulation in the classifier built with all PCHIP coefficients. Similar to selection criteria 2, selection was based on averages of highest ∼10% MDGini values. This criterion was used since the fate of such a chemical species is highly uncertain after the end time of simulation and calls for better scrutiny.

For criteria 2 and 3, MDGini values were obtained from the classifier built using all PCHIP coefficients as it had information pertaining to both function values as well as information about derivatives at a fine time scale. See S1 and S2 Figs, for Box plots for these MDGini values.

### Resolving Significance Over Time for the Selected Species

The five filtered species were further studied by resolving their significance over time. MDGini for these species obtained using the classifier built with all 200s-MA values is shown in Fig 1. Tf-fVIIa-fXa and Tf-fVIIa-fX are most significant around 1200 seconds after clot initiation. Classification accuracies of these individual species using 200s-MA values are tabulated in Table 2. 200s-MA values of Tf-fVIIa-fXa, Tf-fVIIa-fX, IIa, and fIXa-fVIIIa-fX classify better than conventional features (Table 1).

**Table 2:** Classification accuracies (%), mean (SD), for 200s-MA values of selected species. Classification using all 18 200s-MA features of Tf-fVIIa-fXa, Tf-fVIIa-fX, fIXa-fVIIIa-fX, and IIa result in similar accuracies. Classification accuracies of the best 3 and the best feature from each species indicate significance is most localized in Tf-fVIIa-fXa.

Species	All 18	Best 3	Best 1
Tf-fVIIa-fXa	83.96 (0.36)	83.18 (0.47)	78.76 (0.08)
Tf-fVIIa-fX	84.23 (0.40)	82.57 (0.38)	77.32 (0.11)
fIXa-fVIIIa-fX	83.80 (0.47)	78.78 (0.56)	75.26 (0.04)
IIa	84.44 (0.59)	75.86 (0.53)	74.26 (0.04)
fXa	82.07 (0.67)	71.36 (0.81)	53.26 (0.08)

MDGini values from the classifier built with all 200s-MA values were used to choose the subset of best features for each species. Tf-fVIIa-fXa—Tissue factor-factor VIIa-factor Xa; Tf-fVIIa-fX—Tissue factor-factor VIIa-factor X; fIXa-fVIIIa-fX—factor IXa-factor VIIIa-factor X; IIa—activated alpha-thrombin; MDGini—Mean Decrease in Gini index.

A single feature from Tf-fVIIa-fXa classifies with accuracy 78.86%, which suggests that significance of Tf-fVIIa-fXa is best localized in time. This can be seen in Fig 1 as well as in Fig 2. Around 1200 seconds in Fig 2, the mean of the CAD group is outside the 90% quantile of the ACS group. Tf-fVIIa-fX visually behaves in a similar way. This behavior contrasts with a species like fXa (Fig 2).

Among the five species in Table 2, fXa has the lowest accuracy of 82.07%. The best feature of fXa classifies with an accuracy of 53.26% which is marginally better than random guessing (50%). This indicates that all the features of fXa are weak. This is due to a huge overlap between the function values in the two groups (Fig 2). For fXa, conventional features and 200s-MA values classify with an accuracy of about 82% due to the ways in which these weak features interact. Moreover, conventional features of fXa classify marginally better compared to its 200s-MA values due to lack of time information (time to maximum level, rate, etc.,) in 200s-MA values. This suggests that classification accuracies of every species could be further intensified by considering more features based on time information, in particular, time delay.

**Fig 3:**
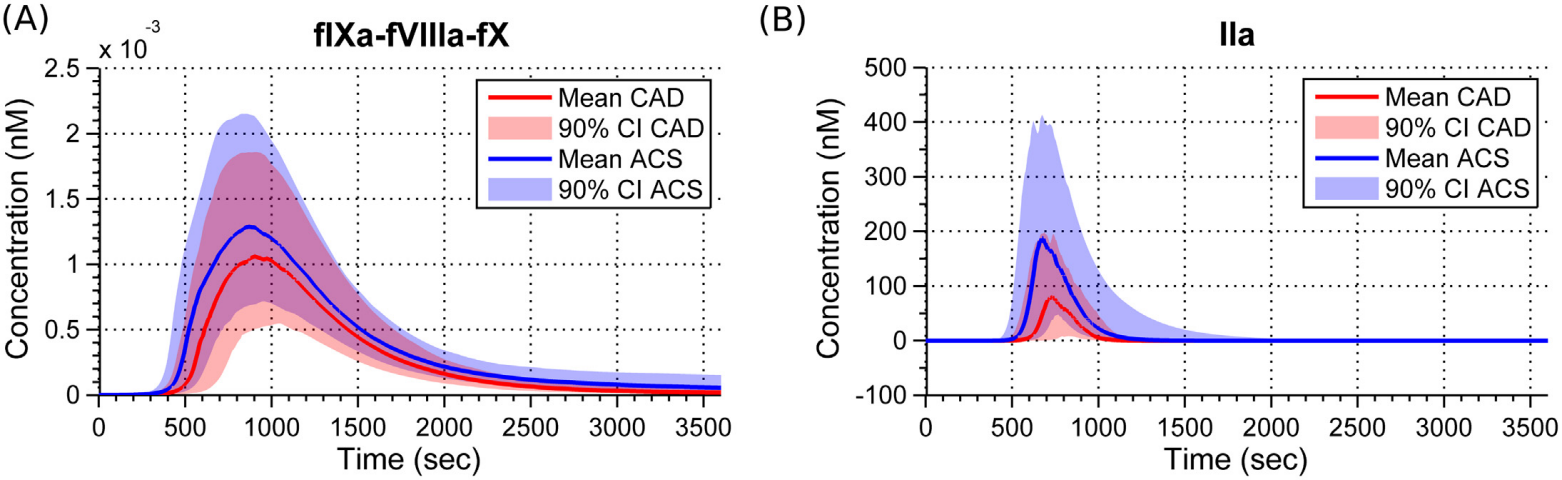
Means and 90% quantiles for fIXa-fVIIIa-fX and IIa simulation profiles in ACS and CAD populations. A: fIXa-fVIIIa-fX profiles show that this species is more long-lived in ACS than CAD cases. B: Though IIa concentration profiles appear to reach zero by 2000 seconds, MDGini suggests that the dynamics between the two groups is most significant during that time. fIXa-fVIIIa-fX—factor IXa-factor VIIIa-factor X; IIa—activated alpha-thrombin; MDGini—Mean Decrease in Gini index.

Concentration profiles for fIXa-VIIIa-fX in the ACS group appear to live longer (Fig 3). Recent studies suggest existence of active circulating particles in blood (long-lived active species) to be a primary mechanism leading to spontaneous clotting in hyper-coagulable blood [30]. As in the case of fIXa-fVIIIa-fX, the computational approach taken here could be tuned to help identify such long-lived differences under various perturbed conditions of the reaction network. Next, we further this discussion using IIa.

200s-MA values of IIa classify better compared to conventional parameters of active thrombin. It is most significant starting at about 1500 seconds by which time its values are in the order of pM. Changes in this region are usually not considered in conventional features. IIa concentration profiles appear to have reached zero by 2000 seconds (Fig 3). However, given the information-theoretic nature of MDGini, it is able to differentiate IIa at regions beyond what is considered as the termination phase of clotting. Given that biological systems are complicated enough where pico moles of certain chemical species could initiate clotting, and perhaps subsequently determine life or death, we do not overlook such a difference here. Regarding precision, we encountered negative concentration values in IIa in the order of 1E-19 (M). The precisions of the numerical solution and PCHIP approximation are possibly inadequate at this scale.

### Classification Performance of a Few Combinations of Species at Specific Times

**Fig 4:**
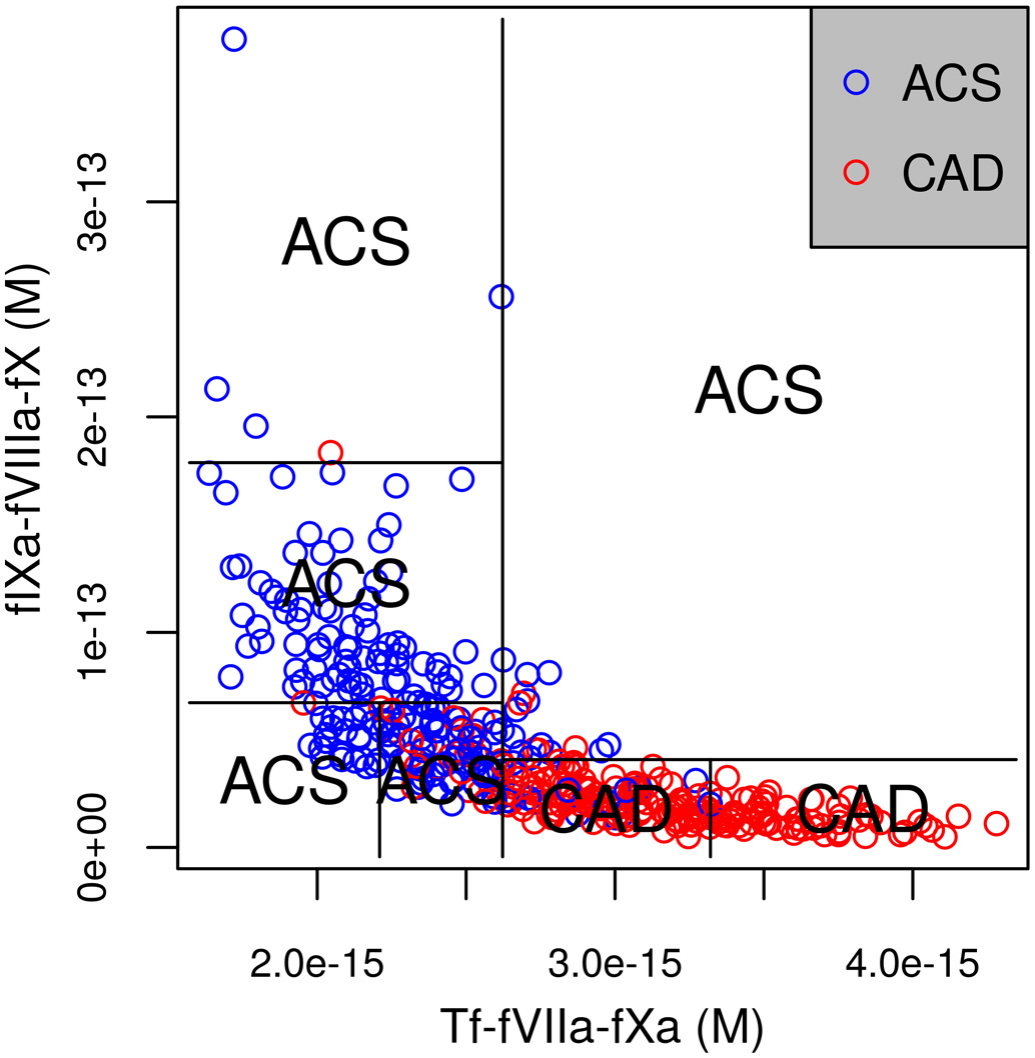
Illustrative decision tree. A single decision tree built with just two of the best 200s-MA features, one each from fIXa-fVIIIa-fX and Tf-VIIa-Xa, is shown. ACS and CAD populations separate well in just those two features. fIXa-fVIIIa-fX—factor IXa-factor VIIIa-factor X; Tf-VIIa-Xa—Tissue factor-factor VIIa-factor Xa; 200s-MA—200-second moving average.

Classification performance of a few combinations of the selected species is shown in Table 3. Average values of Tf-fVIIa-Xa, IIa, and fIXa-fVIIIa-fX at specific times classify with about 87% accuracy. This is better than conventional features or measuring any single species, and is close to using all features considered. For illustration, Fig 4 shows a single decision tree built with just two of the best features from fIXa-fVIIIa-fX and Tf-fVIIa-fXa. Typical of chemical kinetics, the region spanned by the data is localized, suggesting dynamics in a low dimensional manifold. The localized separation of the two groups in 2 dimensions of 200s-MA values is seen in the figure.

**Table 3:** Classification accuracies (%), for classifiers built using combinations of best 200s-MA features. An efficient way to assay the entire system is by measuring three species at three specific time intervals of 200 seconds. Tf-fVIIa-fXa, IIa and fIXa-fVIIIa-fX make the best combination.

Combination	Mean (SD)
Tf-fVIIa-fXa, IIa, and fIXa-fVIIIa-fX	87.16 (0.39)
Tf-fVIIa-fX, IIa, and fIXa-fVIIIa-fX	87.03 (0.40)
Tf-fVIIa-fXa, Tf-fVIIa-fX, and fIXa-fVIIIa-fX	85.58 (0.39)
Tf-fVIIa-fXa, Tf-fVIIa-fX, and IIa	84.90 (0.50)

Tf-fVIIa-fXa at 1400–1600 sec; Tf-fVIIa-fX at 1400–1600 sec; IIa at 1800–2000 sec; fIXa-fVIIIa-fX at 3400–3600 sec; Tf-fVIIa-fXa—Tissue factor-factor VIIa-factor Xa; Tf-fVIIa-fX—Tissue factor-factor VIIa-factor X; IIa—activated alpha-thrombin; fIXa-fVIIIa-fX—factor IXa-factor VIIIa-factor X.

## Discussion

We found high-dimensional feature representations for computational solution profiles and studied how combinations of these features could be used to classify ACS/CAD. We modified and tuned the tools offered by Random Forest to fit our purpose. The species we studied are limited by the species considered in the extrinsic pathway model. We also note that there are at least two other versions of this extrinsic pathway model [31–33].

As our study indicates, we are now in a position to answer the three questions at the beginning of the paper as follows:

1. There exists chemical species that can be used to classify ACS/CAD better than what can be achieved using thrombin and fXa. Our primary list includes Tf-fVIIa-fXa, Tf-fVIIa-X, fIXa-fVIIIa-fX, and IIa. Tf-fVIIa-fXa is the most important species in our list.
2. Conventional features from active thrombin and fXa can be used to classify with an accuracy of 81% and 82.6%. There are better features to characterize the system compared to conventional summary parameters, such as initial conditions (plasma factor composition), which result in a classification accuracy of 88.1%. However, plasma factor composition might not capture many attributes of the reaction network. The entire system, when represented using PCHIP coefficients and 200s-MA values, can be used to classify with accuracies of 88.6% and 88.8%. There could be a lot more going on in the system other than changes in thrombin and fXa. For example, activity of IIa (activated alpha-thrombin) was significantly different beyond the termination phase. Long-term activity of such active species warrants better scrutiny.
3. The entire system could be efficiently assayed by measuring a few combinations of species at well-specified times. For example, concentrations of 3 chemical species, namely IIa, Tf-fVIIa-fXa, and fIXa-fVIIIa-fX, averaged over specific time windows (see Table 3) chosen relative to the time of trigger (Tf), could be used to classify ACS/CAD to an accuracy of about 87.2%. This is a 7.6% improvement in classification accuracy over using the conventional summary parameters of thrombin.

To the best of our knowledge, this is the first study in the literature to find such localized regions labelled in time and in very low dimensions of the state space that could be associated with ACS. Further validation of the classification scheme is contingent upon the availability of more detailed data on these two cases. Such localized and effective combinations, which are also easily measurable, could make good global assays for the thrombin generation system.

## Conclusion

While the random forest technique is a well accepted method for classification in the statistical learning field and has been used in clinical studies, this is the first study in the literature to apply it to classify ACS/CAD using numerical simulations of the thrombin generation system. The approach shows promise in characterizing hyper-coagulability and predicting ACS. Our results open up a way to globally phenotype the thrombin generation system and include specific suggestions for experimental assays to classify ACS/CAD. Currently, measuring some of the recommended chemical species, especially at such low concentration values, may not be practical. However, using models to study combinations of triggers through this approach can reveal measurable chemical species. Moreover, current studies of ACS/CAD classification are restricted to reporting only mean and standard deviation data of plasma factor composition. Wide availability of more raw data would help researchers from diverse fields to study the thrombin generation system and the coagulation cascade.

## Supporting Information

S1 Fig Significance during the entire simulation. Box plots of MDGini values for the PCHIP coefficients for each species. Tf-fVIIa-Xa and Tf-fVIIa-X stand out from the rest of the variables. MDGini values were obtained from the classifier built with all PCHIP coefficients so that their relative importance could be compared for filtering.
(TIFF)

S2 Fig Significance at the end of simulation. Box plots of MDGini values for the PCHIP coefficients taken from the last ten minutes of the simulation. Unlike S1 Fig, many species appear significant based on 5 MDGini values. Average of 25 MDGini values makes fIXa-fVIIIa-fX stand out.
(TIFF)

S1 Dataset Sampled plasma factor composition data. Sampled sets of positive nonzero percentage values of initial conditions for each group (ACS and CAD) from lognormal distributions used in this study. Scaling these with the mean physiological values (see Methods) should give the concentration values of each factor.
(XLSX)
