# Supplementary figures and images for: Random Forests Are Able to Identify Differences in Clotting Dynamics from Kinetic Models of Thrombin Generation
Source: PLoS One. 2016 May 12;11(5):e0153776. doi: 10.1371/journal.pone.0153776 (PMC4865224; doi:10.1371/journal.pone.0153776)

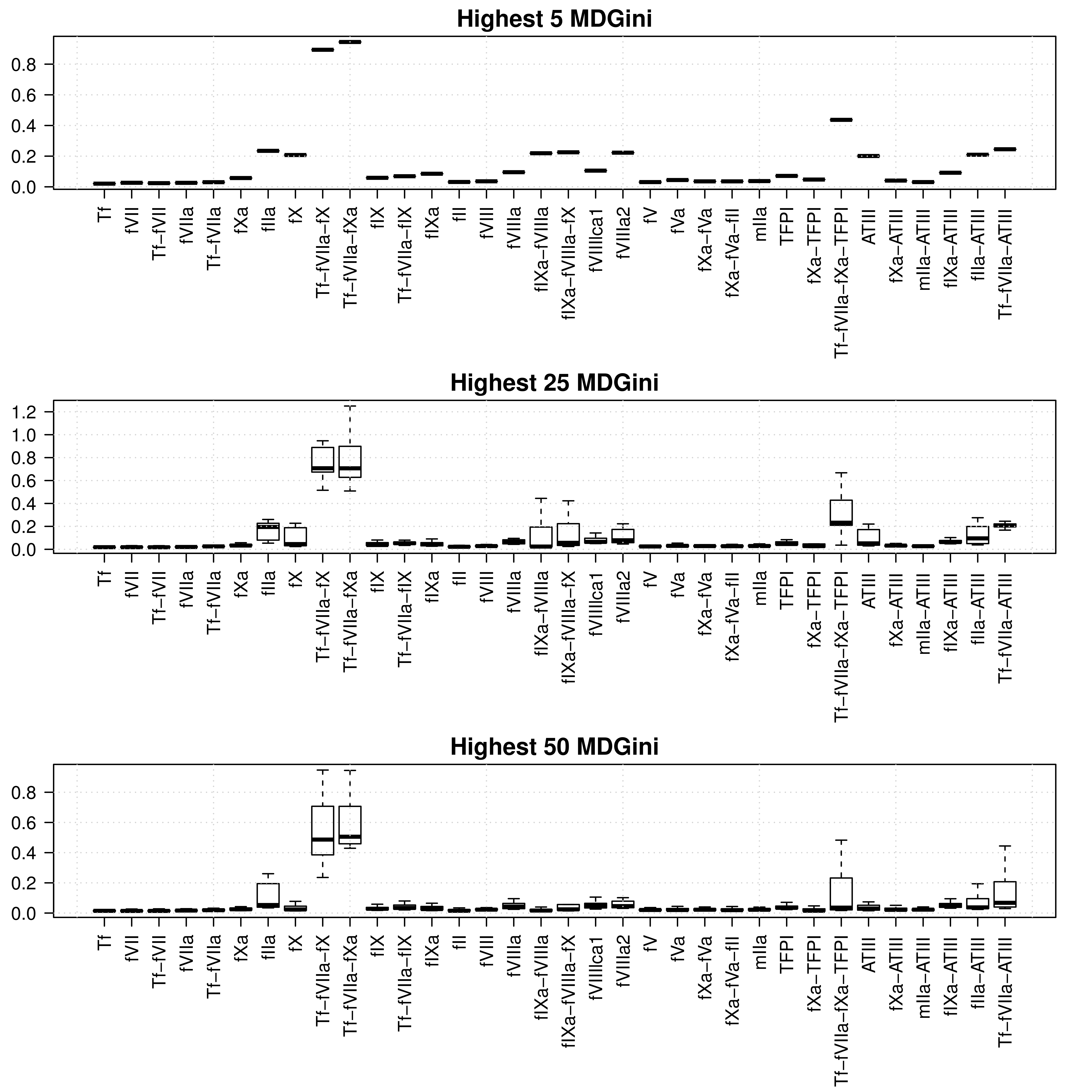

Supplement: S1 Fig — Box plots of MDGini values for the PCHIP coefficients for each species. Tf-fVIIa-Xa and Tf-fVIIa-X stand out from the rest of the variables. MDGini values were obtained from the classifier built with all PCHIP coefficients so that their relative importance could be compared for filtering. (TIFF) [file pone.0153776.s001.tiff]

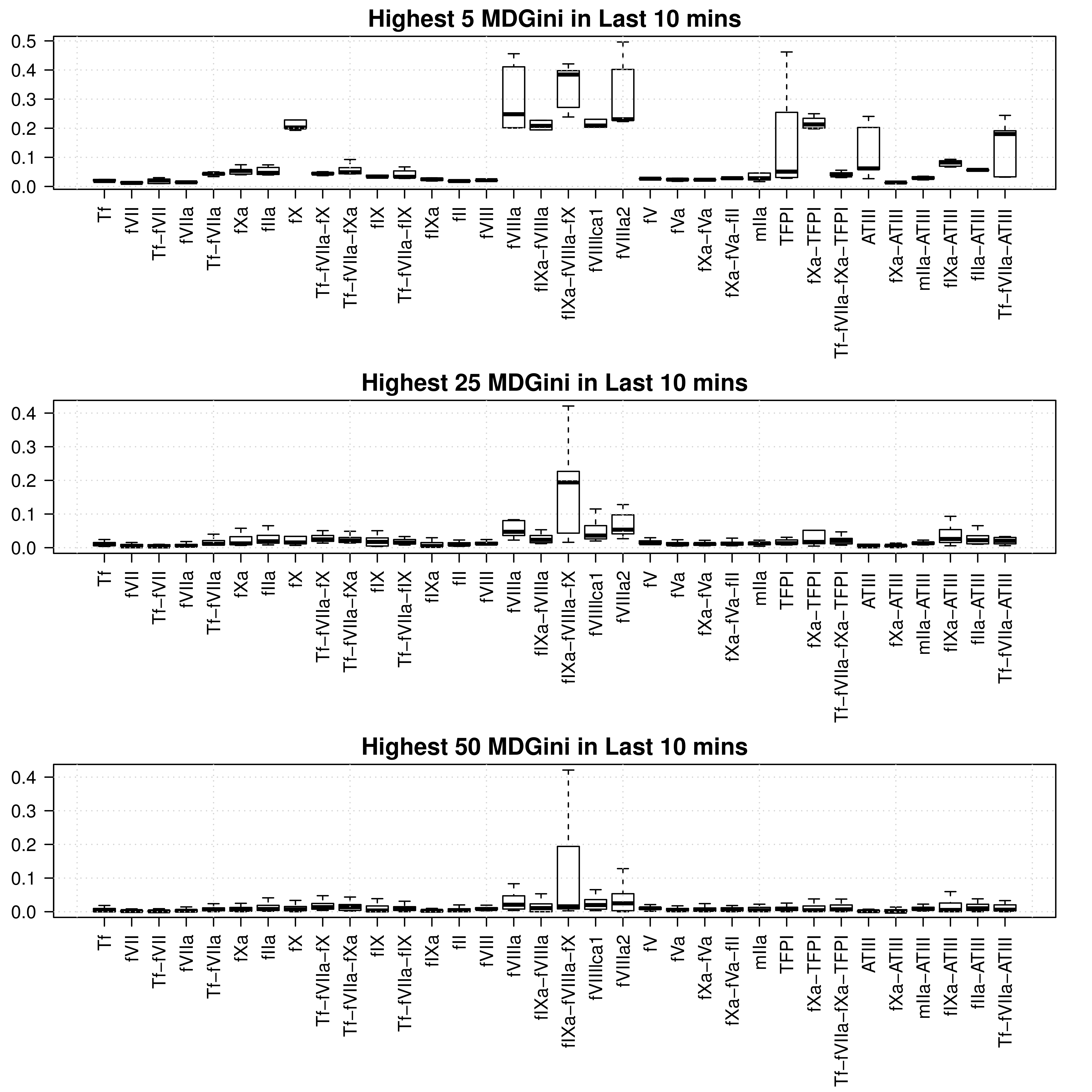

Supplement: S2 Fig — Box plots of MDGini values for the PCHIP coefficients taken from the last ten minutes of the simulation. Unlike S1 Fig, many species appear significant based on 5 MDGini values. Average of 25 MDGini values makes fIXa-fVIIIa-fX stand out. (TIFF) [file pone.0153776.s002.tiff]
